# Change in Time Perception Following the Place of Pre-Existence Technique

**DOI:** 10.3390/ijerph20043509

**Published:** 2023-02-16

**Authors:** Andrea Pintimalli, Joseph Glicksohn, Fabio Marson, Tania Di Giuseppe, Tal Dotan Ben-Soussan

**Affiliations:** 1 Research Institute for Neuroscience, Education and Didactics, Patrizio Paoletti Foundation for Development and Communication, 06081 Assisi, Italy; 2 Department of Criminology, Bar-Ilan University, Ramat Gan 5290002, Israel; 3 The Leslie and Susan Gonda (Goldschmied) Multidisciplinary Brain Research Center, Bar-Ilan University, Ramat Gan 5290002, Israel; 4 Neuroimaging Laboratory, Department of Physiology and Pharmacology, Sapienza University, 00185 Rome, Italy

**Keywords:** Sphere Model of Consciousness, meditation, time perception, sex differences, Place of Pre-Existence

## Abstract

Time perception is closely related to spatial and bodily perception, yet little is known about how this interrelationship is impacted by meditation and biological sex. To examine this, we studied the effects of a stepwise application of three meditation techniques, from focused attention, to open monitoring to non-dual meditation, encompassed in the Place of Pre-Existence technique (PPEt) on the subjective perception of time, space and body using a pre–post research design. A total of 280 participants (mean age = 47.09 years; SD = 10.13; 127:153 males to females) completed the Subjective Time, Self, Space inventory before and after PPEt. Following PPEt, participants perceived time passing as slowing down, while time intensity, relaxation, ‘awareness of space’ and ‘awareness of body’ increased, suggesting heightened mindfulness to these constructs following the training. Awareness of space revealed to be modulated by biological sex and meditation expertise, with males showing a decrease of spatial awareness as a function of meditation expertise while females showed an opposite pattern. The speed and intensity of the experience of time both correlated with body and space awareness. In line with previous studies demonstrating a connection between relaxation and perception of time, a significant correlation was found between relaxation and the subjective experience of the intensity of time. The current results are discussed in the context of the embodied experience of time, and the Sphere Model of Consciousness.

## 1. Introduction

Various meditative techniques have been consistently reported to induce altered and/or higher states of consciousness and, consequently, have become a means for the study of consciousness itself [1,2]. There is an important distinction between higher and altered states of consciousness (ASC), which is generally ignored in the literature: While higher states of consciousness (HSC) are accompanied by improved executive functionality, the opposite is true for many cases of ASC, such as drug-induced ASC. Moreover, electrophysiologically, HSC are accompanied by slower frequency bands and are considered more integrated states [3], while EEG and MRI studies of drug-induced ASC report an opposite trend that could be regarded as fragmentation and consequent deregulation (See [2] for a recent discussion on the matter). Moreover, an important distinction which has been made between general ASC and HSC by Tart [4], is that higher states, in particular, can be associated with superior cognitive functioning or can be more profound than other states; these states can include insights into oneself, insights into others, intuitive understanding of the nature of the universe, or comprehension of an individual’s place in the overall scheme of things. Because most meditations entail a physical focus (e.g., on the breath or other aspects of the bodily functioning), such contemplative techniques may be particularly useful for the study of consciousness with regard to bodily awareness [1]. Accordingly, thanks to the focus on the bodily experience in the present moment, it has been hypothesized that the study of meditation could effectively facilitate detection of bodily correlates of first-person self-awareness by diminishing the “noise” of narrative mental projections of one’s feelings into the past and future [1,5,6]. Bodily experience is, in fact, considered by many to be the basis of general self-awareness itself; for example, Damasio [5] and Panksepp [7] proposed that self-awareness is fundamentally linked to experience of the body. Moreover, several models of consciousness have emphasized the importance of bodily awareness in relation to the experience of time [8] and space [9,10]. For instance, according to Wittmann [8], one’s time perception is related to interoception, which is generally described as a perceptual process through which sensations coming from inside the body are perceived and integrated [11]. In the study, we focused on a specific meditative practice, namely the Place of Pre-Existence technique (PPEt) that combines attention to the body with instructions aiming at diluting the usual temporal points of reference in practitioners’ own autobiographical memory. As we shall see, the PPE is a stepwise application of three meditation techniques (from Focused Attention to Open Monitoring to Non-Dual Meditation—FAM, OMM, NDM, respectively).

**Figure 1:**
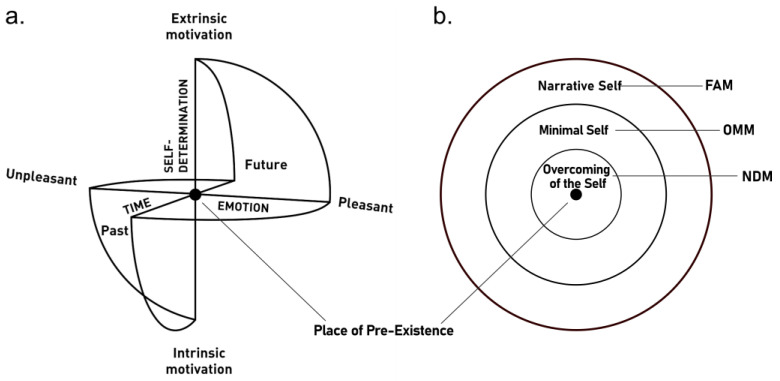
(**a**) Sphere Model of Consciousness (SMC) axes and polarities. (**b**) Layers of the Sphere Model of Consciousness, from the more external to the more internal. Each layer corresponds to one state of the self (narrative self, minimal self, overcoming of the self) and it is associated with a specific type of meditation (focused attention meditation, open monitoring meditation, non-dual meditation). Adapted from Paoletti and Ben-Soussan [6,12]. FAM = focused attention meditation; OMM = open monitoring meditation; NDM = non-dual meditation.

PPEt is derived from the Sphere Model of Consciousness (SMC [6,12]), a neuro-phenomenological model applied also in educative contexts [13,14]. The SMC is based on the main elements thought to characterize conscious experience, namely, time, emotion, and self-determination (Figure 1a). In the SMC, these three elements’ polarities are depicted as intersect axes in a spherical representation of the dynamics of conscious experience. According to the model, all possible subjective experiences can be placed along the three axes in a phenomenological depiction of time (past–future), emotion (pleasant–unpleasant), and self-determination (intrinsic–extrinsic motivation). The SMC’s aptly named ‘place of pre-existence’, visually located at the center of the sphere, is considered the state in which one may experience ‘overcoming of the self’.

In fact, in the SMC model three distinct forms of self are depicted as concentric rings surrounding a central point (Figure 1b). The narrative self (autobiographical memories, projections into the future, conceptual contents, and continuous awareness of personal identity), the minimal self (awareness of the body as a sensorimotor unit, embodied selfhood anchored in the “here and now,” and non-conceptual contents), and the overcoming of the self (disappearance of all or almost all sense of self with possible preserved capacity to observe and report subjective experiences). Recently, these three states of self have been found to correspond to the foci of three specific types of meditation: focused attention meditation (FAM), open monitoring meditation (OMM), and non-dual meditation (NDM) [2], respectively.

The Place of Pre-Existence technique (PPEt), therefore, was designed to attempt to dilute participants’ temporal points of reference (FAM), while oral instructions are consequently provided to elicit experiences of dissolution of bodily boundaries (OMM), usually associated in practitioners’ experience with the dissolution of spatial boundaries (NDM) [15]. According with the found correspondence between meditations’ foci and Self types, participants are hypothesized to shift from narrative self to minimal self to overcoming of the self [2]. The PPEt design is based on the idea that, thanks to the steps through FAM, then OMM and finally NDM, the practitioners can be taken to an emotionally neutral feeling regarding the past and the present. The final part of the technique, after the NDM is to invite the participants to conceive the future they desire from ‘the center of the sphere’, a state in which they can be generally unaffected by autobiographical experiences [16]. Thus, in the PPEt, the metaphor of an empty place [17] is used to describe the sense of detachment that exemplifies entering the center of the sphere towards the state of non-dual ‘overcoming of the self’. 

A recent study related to PPEt examined the experience of 481 participants before and after an intensive 3-day PPEt training [16]. The participants completed a semi-structured self-report questionnaire regarding their experience. The results revealed a pre–post PPEt training shift from predominant influence of the mental aspect to a spatial predominant influence on participants’ experience. Moreover, while before training their experience of space did not appear to influence other dimensions, following training, the experience of the space dimension affected their self-determination, particularly problem-solving ability, aspirations, and release from conditioning. This finding suggests that a shift occurred away from the narrative self, in which the mental component is more prominent, toward the minimal self, in which the bodily and spatial dimensions are more prominent. This finding of a change in the phenomenology of consciousness supports the notion that PPEt training impacts the spatial dimension of experience over other dimensions. 

However, the current state of knowledge related to neuro-phenomenology of the Self is under-developed with regard to its different dimensions and how they are impacted by different meditative paradigms [18]. In addition, it is unclear how biological sex may or may not factor in the experience of shifting between the selves and how. Consequently, in the current study we sought to examine the possible relationship between the dynamic change regarding the experience of time, space and the bodily self, and their eventual correlation with biological sex and meditation expertise variables following the PPEt. To this aim, we chose to utilize the previously used Visual Analogue Scale (VAS, [19]) and questions from the Subjective Time, Self, Space Inventory (STSS; [20,21], see methods Section 2.3. Measures for additional details). 

### Meditation, Time Perception, and Biological Sex

The subject of interrelationship between meditation, time and biological sex is of particular interest, both conceptually and in light of past investigations [22,23,24] on meditative practices in our lab [2,6,12,16,25,26]. In those investigations, several sex-related meditation training effects were found in terms of the subjective estimation of time and electrophysiology. Glicksohn and Ben-Soussan [26] used a pre–post research design to assess the effects of one month of daily Quadrato Motor Training (QMT) a SMC-based structured movement meditation consisting of sequences of motor acts in response to verbal directions on time production (TP). In contrast to the verbal training control group (identical cognitive training with no overt motor component, and only verbal responses), longer and more accurate TP in females was found following 1 month of intensive QMT in a dyslexic group, while the opposite trend was observed in control females, possibly as a result of QMT-induced increased attention. These findings suggest that the combination of motor movement and mindfulness training included in QMT has varying effects based on sex and dyslexic status.

In addition, in a previous study on the relationship between QMT, electrophysiology, and reflectivity (self-examination of one’s conscious thoughts and feelings accompanied by inhibition of habitual thought and behavior), QMT’s sex-related effects among 37 individuals (20 men) was examined. QMT increased reflectivity (measured as the interpolation between correct responses and slower reaction time within a spatial cognition task) in both sexes, while a sex-dependent variation in functional connectivity was discovered. Whilst theta (4–7 Hz) and alpha (8–12 Hz) intra-hemispheric coherence was increased in females after QMT, the reverse pattern was observed in males [25]. These findings are consistent with the hypothesis that neural efficiency in males manifests in local cortical oscillations, whereas neural efficiency in females manifests in the functional coupling of several brain regions, as measured by EEG coherence [26]. Moreover, in a preliminary investigation into the electrophysiological effects of the SMC-based OVO Whole-Body Perceptual Deprivation chamber (OVO-WBPD, named for its shape: ovo is Latin for egg; [27,28]), we also found sex-related EEG differences. More specifically, a predominant R > L alpha asymmetry was found for male participants while a predominant L > R asymmetry was found for female participants [28].

In addition to the sex-related findings, this study further discovered that the OVO-WBPD elicited synesthetic experiences of cognitive dedifferentiation [29] involving diverse sensorial dimensions. Interestingly, the synesthetic experiences involved space and time less than other dimensions, consistent with Fabbri and colleagues’ [30] findings that the spatio-temporal association is influenced by movement more than visuospatial factors.

Given the preliminary indications in the literature and in our lab’s previous research that meditative practices may induce a shifting between different types of Self, even leading to the overcoming of any sense of self; that the process leading to shifts between the diverse states of Self may be different depending on biological sex; and that level of expertise may affect the effect of the meditative practices, the aims of the present study were threefold: 1) to explore with a pre–post perspective how the PPEt, through the “collapse” of SMC axes (time, emotion, self-determination) within the subjective perception of meditative practitioners, might induce a shift between the types of Self and how this affects, time perspective; 2) to examine whether are there sex-related effects associated with PPEt; and 3) to examine whether are there differences in the impact of PPEt based on participants’ previous meditation expertise, and how such expertise correlates with other variables. 

Regarding the design, we opted for a pre–post investigation because supposedly the collapsing of SMC axes through the PPEt would elicit a non-dual state which is characterized by ineffability, thus making it eventually impossible for participants to report their phenomenological experience during the practice [2].

## 2. Materials and Methods

### 2.1. Participants and General Procedure

A total of 301 participants were originally recruited for this study, and 280 were included in the final data analysis (age range = 21~73; mean age = 47.09 years; *SD* = 10.13; 127:153 males to females). For the 21 participants ultimately excluded from the data analysis, one or more of the following reasons applied: lack of completion of at least 50% of the questionnaire, provision of incomprehensible responses, or being a non-native Italian speaker (this exclusion criterion was intended to preserve response reliability because the questionnaire instructions were provided in Italian). Despite previous experience with meditative practice among some of the participants, this particular technique was new to all of the participants. The study was approved by the Ethics Committee at Bar-Ilan University. All participants provided signed informed consent and took part in a group meditative session on the Place of Pre-Existence technique (PPEt, [16]) filling the questionnaires before and after the PPEt.

### 2.2. Place of Pre-Existence Technique (PPEt)

**Figure 2:**
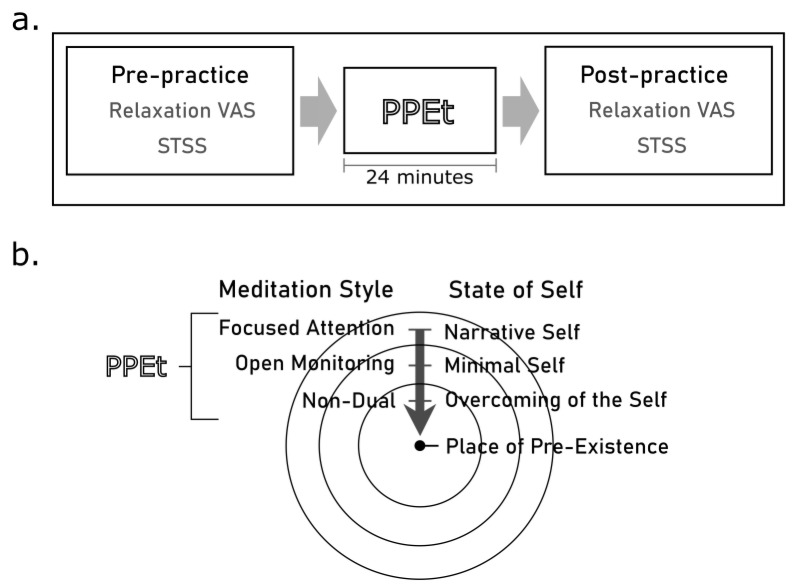
(**a**) Experimental protocol. A measurement for relaxation on a visual analogue scale (relaxation VAS) and items from the Subjective Space Time and Self inventory (STSS) were collected before and after 24 min of practice of Place of Pre-Existence technique (PPEt). (**b**) Layout of the PPEt practice showing the progressive steps involving different styles of meditation (left) required to promote the transition across selves (right) towards the Place of Pre-Existence.

The PPEt is a guided meditation that aims to bring practitioners to a neutral feeling regarding the past and the present, allowing conception of the future from ‘the center of the sphere’, a state in which one can be generally detached by autobiographical events. PPEt employs the metaphor of an empty space [17] to depict this level of detachment, which is a manifestation of the overcoming of the self. This metaphor is supposed to enable the practitioner to transcend a dualistic understanding of emotion (pleasant vs. unpleasant) and time (past vs. future) [12]. In this context, it is thought that deliberate mental silence helps to move one closer to the state represented by the center of the sphere [16,31,32,33], through a path starting with focused attention meditation (FAM), continuing with open monitoring meditation (OMM), and ending with non-dual awareness (ND). More specifically, the participants practiced the PPEt by listening to a recorded audio guide in which the narrator provided directions to facilitate deep relaxation, to enable retracing one’s autobiographical history by recalling episodes from one’s life and detaching from them (focused attention meditation), to encourage letting go of bodily boundaries (open monitoring meditation), and to reach a state of non-duality (non-dual meditation). The meditation session was conducted in a group setting, where participants were seated on a chair. Each session lasted 24 min (see Figure 2). 

### 2.3. Measures

**Figure 3:**
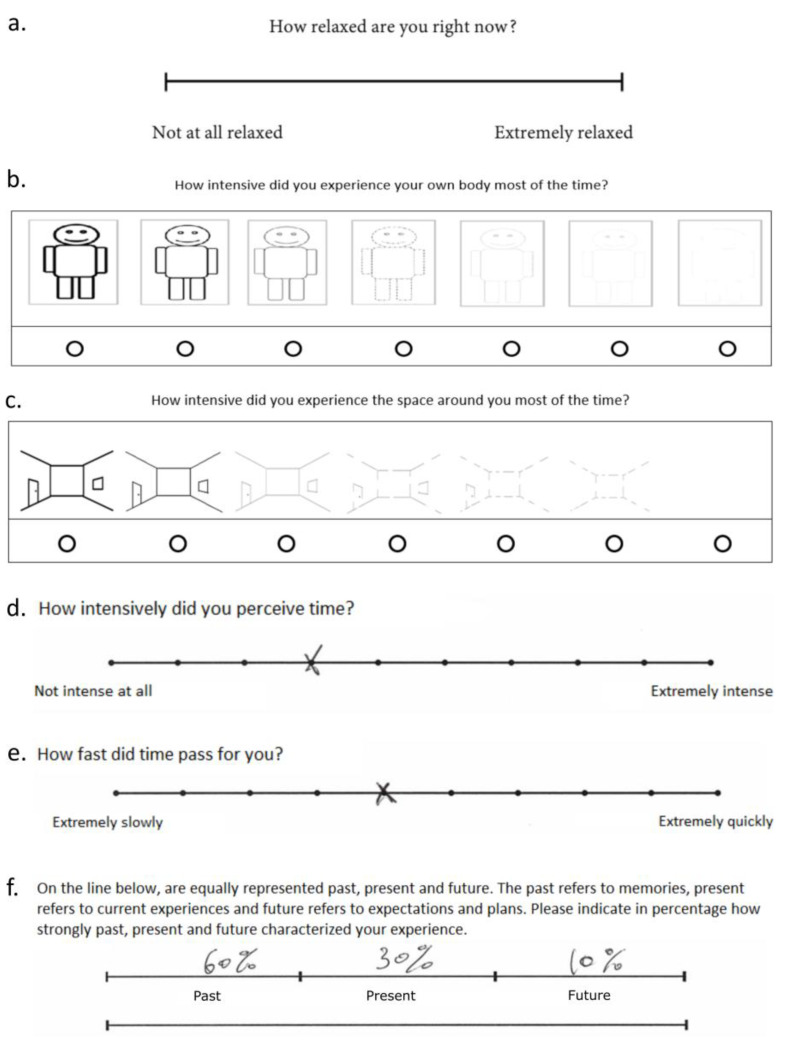
(**a**) VAS used to assess relaxation; (**b**) scale adopted to assess body awareness; (**c**) scale adopted to assess space awareness; (**d**) scale adopted to assess time intensity; (**e**) scale adopted to assess time speed; (**f**) scale adopted to assess time perspective: a possible answer by a participant is provided for illustration purposes in (**d**–**f**). Tests adapted from Pfeifer et al., 2016 [20].

First, relaxation was assessed using a visual analogue scale (VAS, [19]) consisting of a 13 cm horizontal line whose opposite ends were labeled “not relaxed at all” and “extremely relaxed” (Figure 3a). Participants were asked to indicate their current level of relaxation. Their VAS scores were transformed into a 0-to-1 scale in order to perform statistical analysis, coding the left extremity of the VAS as 0 and the right extremity of the VAS as 1. Subsequently, questions from the Subjective Time, Self, Space inventory (STSS; [20,21]) were administered. Participants’ experience of body awareness (Figure 3b) and spatial awareness (Figure 3c) was assessed via 7-point Likert scales with visual cues representing different levels of awareness. While on the original scale, “1” was related to the highest awareness score and “7” to the lowest, during data analysis the scores were reversed to make them more intuitively relatable such that “1” represented the lowest and 7 the highest awareness score. Regarding the subjective awareness of time passing, two 10-point Likert scale items were presented: (1) time intensity ([20,21] Figure 3d), soliciting a self-report of how aware one is of the passage of time in terms of perceived intensively, ranging from “not intensive at all” to “extremely intensive”; and (2) time speed (Figure 3e), soliciting a self-report of the speed at which time has being perceived to pass during the technique, ranging from “extremely slowly” to “extremely quickly”. Finally, participants were asked to express in percentages how much the past, present, and future characterized their current experience [20], with all three parts adding up to 100% (Figure 3f).

As we were interested in observing what changes (if any) would occur in the indices following the practice related to previous meditation experience, an additional index regarding the participants’ meditation expertise, named total meditation hours was calculated (years of practice × days each week of practice × number of weeks per year × minutes for each session)/60). 

### 2.4. Statistical Analysis

Raw data from relaxation, body awareness, space awareness, time intensity, time speed, and prominence of time (past, present and future) indices collected before and after the PPEt practice were used for analysis. Then, index scores obtained before PPEt practice (pre) were compared to those obtained after PPEt practice (post). To address pre–post changes, each index was modeled and entered in a repeated measures analysis of covariance (rmANCOVA) using the Satterthwaite approximation for degrees of freedom [34] with training (pre vs. post) as a within-participants variable, sex (male vs. female) as a between-participants variable, and total meditation hours as a continuous covariate. 

Following reviewers’ suggestion, we also ran an additional multivariate analysis of covariance (MANCOVA) with relaxation, body awareness, space awareness, time intensity, and time speed as dependent variables, assessment (re vs. post) as within-subject variable, gender (males vs. females) as a between-subject variable and total hours of meditation as a covariate. 

Subsequently, correlations between variables were computed in order to determine whether and how participants’ total meditation hours and index changes were related. Pre-training, post-training, and delta (computed as post minus pre) scores were considered for correlation analysis using Pearson correlations. Lastly, correlations between delta scores and the indices were computed. The false discovery rate correction (fdr) for multiple comparisons was applied to the 30 observed *p*-values adopting the Benjamini and Hochberg method [35] All *p*-values in the results section and in the graphs are fdr corrected. As explained previously, the indices used in the current study were collected via VAS or interval scales, with scores often showing skewness towards one of the two extreme values of the scales, especially in the post training. Thus, the normality assumption was not always met. Nonetheless, the use of parametric statistics on non-normally distributed data does not represent a major concern in the present context, given the robustness that parametric analysis has shown in studies adopting a large sample size, such as the current one [36,37].

All analyses and graphs were computed using RStudio (RStudio: Integrated Development for R. RStudio, PBC, Boston, MA, USA). Data were modeled in linear mixed models using the *lmer* function from the lme4 package, and rmANCOVAS and Bonferroni-corrected comparisons were computed with the *anova_stats* and *emmeans* functions from the *sjstats* and *emmeans* package, respectively. Correlations were computed using the *cor.test* function from the *base* package and plots were generated using *ggviolin* and *ggscatter* functions from the *ggpubr* package.

## 3. Results 

### 3.1. rmANCOVA

#### 3.1.1. Relaxation

**Figure 4:**
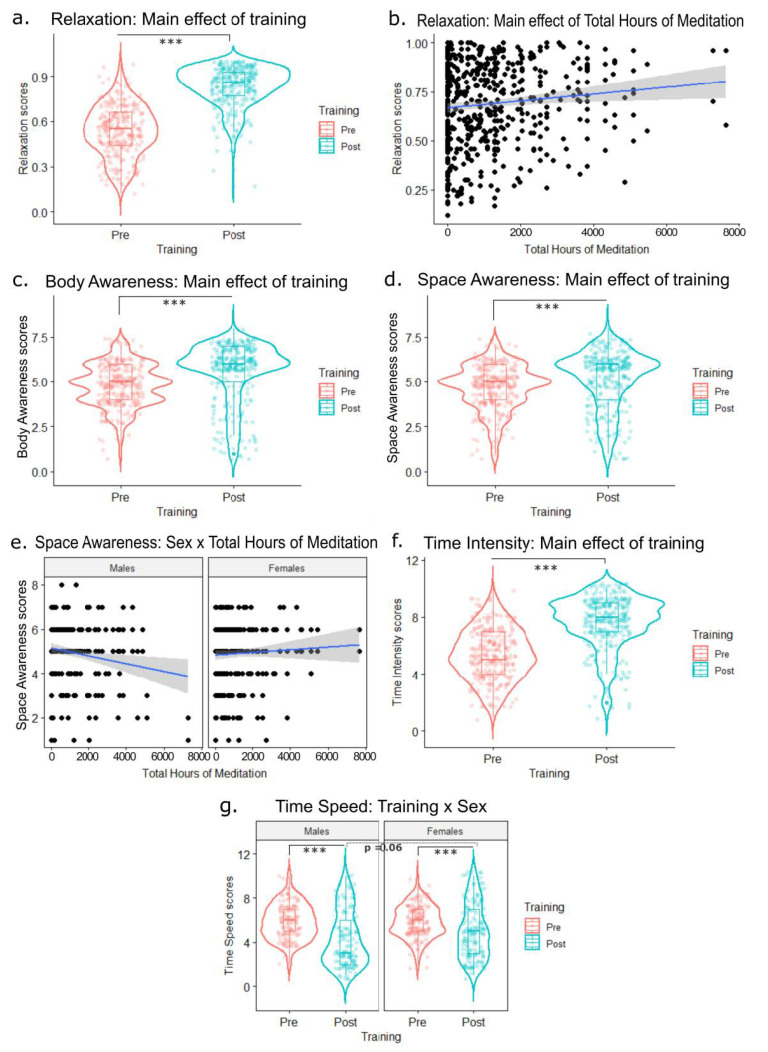
(**a**) Main effect of training (pre vs. post) on relaxation scores. (**b**) Main effect of total meditation hours (covariate) on relaxation scores. (**c**) Main effect of training (pre vs. post) on body awareness scores. (**d**) Main effect of training (pre vs. post) on space awareness scores. (**e**) Interaction of sex (males vs. females) x total meditation hours on space awareness scores. (**f**) Main effect of training (pre vs. post) on time intensity scores. (**g**) Interaction of training (pre vs. post) × sex (males vs. females) on body awareness scores. *** = *p* < 0.001.

A significant main effect of training [F(1,272.99) = 542.8; *p* < 0.001; η_p_2 = 0.59] was observed, showing that post-training relaxation scores were significantly higher following training (pre = 0.53 vs. post = 0.83; Figure 4a). Also, the main effect of the total meditation hours covariate was significant [F(1,277.65) = 9.02; *p* < 0.01; η_p_2 = 0.02], indicating that greater expertise in previous meditation practice was associated with a greater level of relaxation (Figure 4b). No other significant main effects or interactions were observed (*p* > 0.50). Exploring the individual trends, 95.95% of participants (N = 261) reported increased relaxation across time points, 3.31% of participants (N = 9) reported decreased relaxation across time points, and 0.73% of participants (N = 2) reported no change in relaxation across time points.

#### 3.1.2. Body Awareness

A significant main effect of training [F(1,276) = 29.78; *p* < 0.001; η_p_2 = 0.06] was observed, showing that post-training body awareness scores were significantly higher (pre = 4.80 vs. post = 5.59; Figure 4c). No other significant main effects or interactions for body awareness were observed (all ps > 0.38). Exploring the individual trends, 67.14% of participants (N = 188) reported increased body awareness across time points, 17.14% of participants (N = 48) reported decreased body awareness across time points, while 15.71% of participants (N = 44) reported no change in body awareness across time points.

#### 3.1.3. Space Awareness

A significant main effect of training [F(1,276) = 14.88; *p* < 0.001; η_p_2 = 0.03] was observed, showing that post-training space awareness scores were significantly higher than pre-training scores (pre = 4.68 vs. post = 5.16; Figure 4d). A significant sex × total meditation hours interaction was observed [F(1,276) = 6.12; *p* < 0.05; η_p_2 = 0.01]. The impact of total meditation hours affected males and females differently, indicating that space awareness scores decreased with the total meditation hours among males, whereas they increased with total hours of meditation in females (Figure 4e). No other significant main effects or interactions were observed (all ps > 0.15). Exploring the individual trends, 58.92% of participants (N = 165) reported increased space awareness across time points, 23.21% of participants (N = 65) reported decreased space awareness across time points, while 17.85% of participants (N = 50) reported no change in space awareness across time points.

#### 3.1.4. Time Intensity

A significant main effect of training [F(1,273.97) = 150.13; *p* < 0.001; η_p_2 = 0.26] was observed, indicating that post-training time intensity scores were significantly higher than pre-training scores (pre = 5.38 vs. post = 7.67; Figure 4f). No other significant main effects or interactions were observed (all ps > 0.18). Exploring the individual trends, 85.2% of participants (N = 236) reported increased time intensity across time points a, 7.94% of participants (N = 22) reported decreased time intensity across time points, while 6.85% of participants (N = 19) reported no change in time intensity across time points.

#### 3.1.5. Time Speed

A significant main effect of training [F(1,275.71) = 32.16; *p* < 0.001; η_p_2 = 0.06] was observed, indicating that post-training time speed was significantly slower (pre = 5.86 vs. post = 4.64). A significant training × sex interaction was observed [F(1,275.71) = 4.09; *p* < 0.05; η_p_2 = 0.01]. In this regard, males and females did not differ in time speed in the pre-training phase (pre-male = 5.86; pre-female = 5.86; *p* = 1) while the difference between males and females in post-training time speed scores showed a trend towards significance (*p* = 0.063) of a greater slowdown of time passage following training for males (post-male = 4.33; post-female = 4.96; Figure 4g). No other significant main effects or interactions were observed (all ps > 0.19). Exploring the individual trends, 25.8% of participants (N = 72) reported increased time speed across time points, 61.29% of participants (N = 171) reported decreased time speed across time points, and 12.9% of participants (N = 36) reported no change in time speed perception across time points.

#### 3.1.6. MANCOVA

Multivariate results showed a significant main effect of assessment (*p* < 0.001) and total hours of meditation (*p* < 0.01), while the main effect of gender and the assessment × gender interaction were not significant (*p* = 0.17 and *p* = 0.25, respectively).

Univariate results showed a significant main effect of assessment for all the dependent variables (all *p* > 0.001), a marginally significant main effect of gender for time speed only (*p* = 0.047), and a significant main effect of total hours of meditation for relaxation (*p* < 0.001).

The results from the multivariate analysis were almost identical to the results obtained through running separate rmANCOVAs. The only difference is the significance of the main effect of gender in determining time speed scores. However, this main effect was not significant when post hoc tested in a univariate ANCOVA with gender as between variable and total hours of meditation as covariate (*p* = 0.07).

### 3.2. Prominence of Time: Past, Present, Future

#### 3.2.1. Past

**Figure 5:**
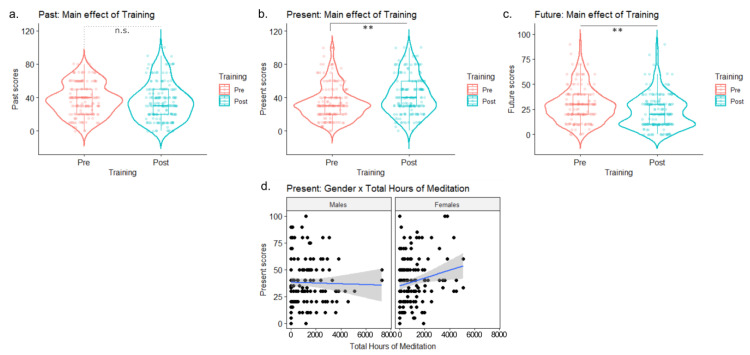
(**a**) Main effect of training (pre vs. post) on past time perspective scores. (**b**) Main effect of training (pre vs. post) on present time perspective scores. (**c**) Main effect of training (pre vs. post) on future time perspective scores. (**d**) Plot of the sex x total hours of meditation interaction on present time perspective scores. Significance levels: ** = *p* < 0.01; n.s. = *p* > 0.05.

No main effects or interactions were observed (all *p* > 0.11). Exploring the individual trends, 33.97% of participants (N = 53) reported increased past scores across time points, 55.77% of participants (N = 87) reported decreased past scores across time points, and 10.25% of participants (N = 16) reported no change in past time perspective across time points (Figure 5a).

#### 3.2.2. Present

A significant main effect of training [F(1,164.22) = 6.92; *p* < 0.01; η_p_2 = 0.02] was observed, indicating present time perspective scores were significantly higher following PPEt (pre = 5.50 vs. post = 6.27; Figure 5b). A significant sex × total meditation hours interaction was observed [F(1,162.47) = 3.94; *p* < 0.05; η_p_2 = 0.01]. Total meditation hours affected males and females differently, indicating that present scores increased with the total meditation hours among female participants, whereas they remained stable across total meditation hours among male participants (Figure 5d). No other significant main effects or interactions were observed (all ps > 0.13). Exploring the individual trends, 58.33% of participants (N = 91) reported increased present scores across time points, 27.56% of participants (N = 43) reported decreased present scores across time points, and 14.1% of participants (N = 22) reported no change in present time perspective across time points.

#### 3.2.3. Future

A significant main effect of training [F(1,169.02) = 7.96; *p* < 0.01; η_p_2 = 0.02] was observed, indicating post-training future time perspective scores were significantly lower following PPEt (Pre = 29.1 vs. Post = 22.1; Figure 5c). No other significant main effects or interactions were observed (all ps > 0.13). Exploring the individual trends, 29.87% of participants (N = 46) reported increased future scores across time points, 55.19%of participants (N = 85) reported decreased future scores across time points while 14.93% of participants (N = 23) reported no change in Future time perspective across time points.

### 3.3. Correlations between Indexes of Interest between Time Points

**Figure 6:**
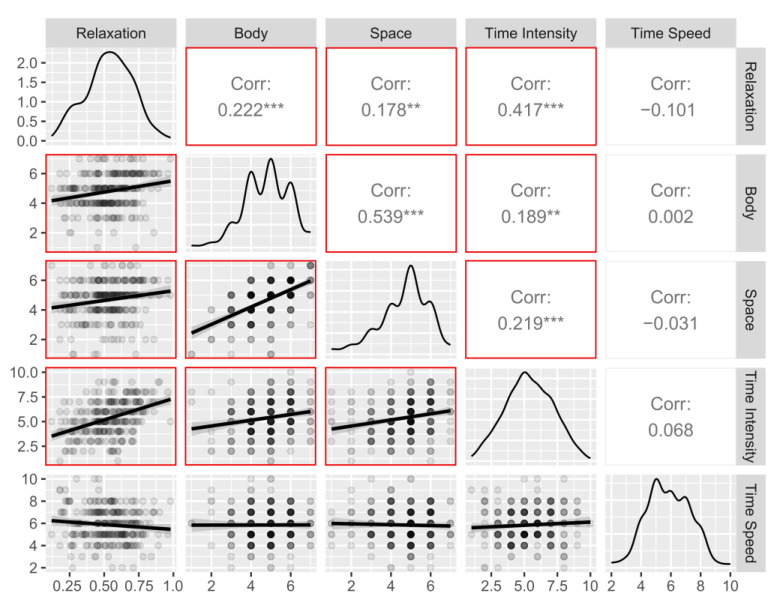
Relationship between pre-training indices: scatterplots, distributions, and Pearson’s correlations. Significant correlations are framed by the red square. Body = Body Awareness, Space = Space Awareness. *** = *p* < 0.001, ** = *p* < 0.01.

**Figure 7:**
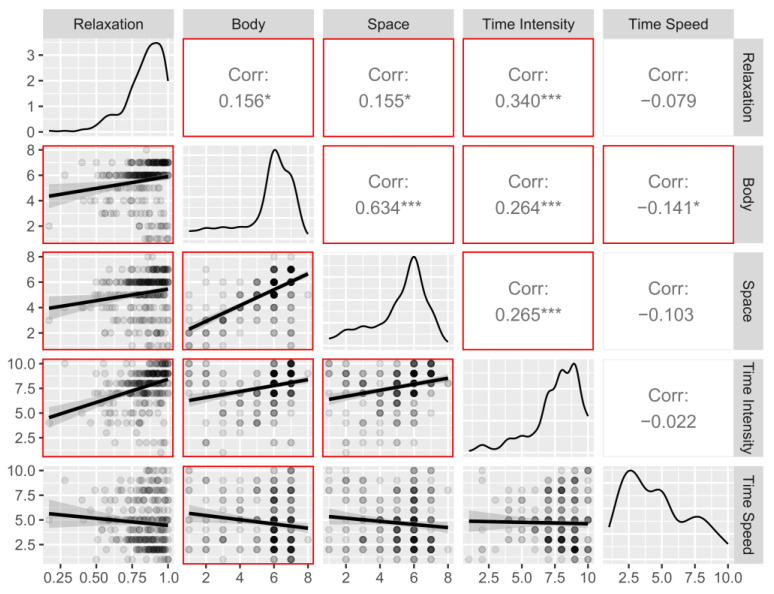
Relationship between post-training indices: scatterplots, distributions, and Pearson’s correlations. Significant correlations are framed by the red square. Body = Body Awareness, Space = Space Awareness. *** = *p* < 0.001, * = *p* < 0.05.

Consistent positive correlations between relaxation, body awareness, space awareness and time intensity were observed when considering pre- and post-training scores separately (Figure 6 and Figure 7). In detail, body awareness and space awareness showed the strongest correlations at both time points (pre: r = 0.53, *p* < 0.001; post: r = 0.63, *p* < 0.001). Time intensity was also correlated with body awareness (Pre: r = 0.19, *p* < 0.01; Post: r = 0.26, *p* < 0.001) at both time points, as well as with space awareness (pre: r = 0.21, *p* < 0.001; post: r = 0.26, *p* < 0.001). Relaxation resulted in a significant correlation with time intensity at both time points (pre: r = 0.41, *p* < 0.001; post: r = 0.34, *p* < 0.001) and also with body awareness (pre: r = 0.22, *p* < 0.001; post: r = 0.15, *p* < 0.05) and space awareness (pre: r = 0.17, *p* < 0.01; post: r = 0.15, *p* < 0.05). Interestingly, perception of time speed showed no significant correlations to other indices during the pre-training period (all ps > 0.12), but it showed a significant correlation with body awareness post-training (r = −0.14, *p* < 0.05; all other ps > 0.11).

**Figure 8:**
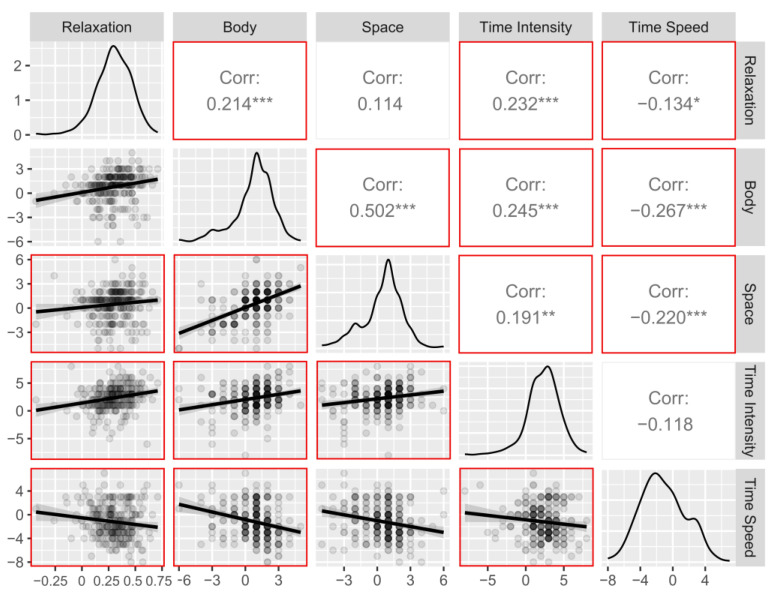
Relationship between indices’ pre- to post-training change (delta) scores: scatterplots, distributions, and Pearson’s correlations. Significant correlations are framed by the red square. Body = Body Awareness, Space = Space Awareness. *** = *p* < 0.001, ** = *p* < 0.01, * = *p* < 0.05.

When considering changes across time points in terms of delta scores, a strong correlation was observed between changes in body awareness and space awareness (r = 0.50; *p* < 0.001), indicating that an increase in one index was associated with an increase in the other (Figure 8). Changes in time intensity were significantly correlated with changes in body awareness (r = 0.24; *p* < 0.001) and changes in space awareness (r = 0.19; *p* < 0.01), indicating that increases in time intensity were associated with increases in body awareness and space awareness scores across time points. Changes in relaxation were correlated with changes in body awareness (r = 0.21; *p* < 0.001), time intensity (r = 0.23; *p* < 0.001) and time speed (r = −0.13; *p* < 0.05), but not with changes in space awareness (*p* > 0.08), indicating that increases in relaxation were associated with increases in body awareness, time intensity and time speed scores across time points. Finally, changes in time speed were significantly negatively correlated with body awareness (r = −0.26; *p* < 0.001) and space awareness (r = −0.22; *p* < 0.001), while its correlation with time intensity was not significant (*p* = 0.07), indicating that slower perception of time speed was associated with increased body awareness, space awareness, and relaxation scores across time points.

## 4. Discussion

The study’s threefold objectives were: (1) utilizing a pre-post design, to explore how the PPEt, through the “collapse” of SMC axes (time, emotion, self-determination) within the subjective perception of meditative practitioners, might induce a shift between the types of self and how this affect time perspective; (2) to examine whether there are sex-related effects associated with PPEt; and (3) to examine whether there are differences in the impact of PPEt based on participants’ previous meditation expertise, and how such expertise correlates with other variables.

### 4.1. Collapsing the Phenomenological Features of Experience

We found that after the PPEt experience, there were significant increases in participants’ reported levels of relaxation, time intensity, body awareness, and space awareness, along with a significant decrease in perception of time speed. The observed increased perception of time intensity after PPEt practice suggests that participants tended to attribute more “weight” to their time focusing on diluting their temporal points of reference, following the technique’s instructions, than before practicing. The increased body awareness and space awareness identified after PPEt also reflect an increase in how vivid one’s perception is of the body and its connection with the spatial environment, which is consistent with previous studies on the attenuation of bodily boundaries [8,16,38].

Moreover, relaxation was strongly positively correlated with perception of time intensity (in pre-practice, in post-practice, and when looking at changes between time points) indicating that greater relaxation was associated with a more intense perception of the passage of time. Changes in relaxation ratings were also negatively correlated with changes in time speed but, despite being significant, this correlation was weaker than the one with time intensity. In addition, relaxation was found to be consistently positively associated with increased body awareness (in pre-practice, in post-practice, and when looking at changes between time points), suggesting that the bodily experience is strictly related to levels of relaxation. Relaxation also significantly correlated with space awareness before and after practice, but no correlation was found when looking at the changes between time points, suggesting that these two indices are strictly related but do not necessarily vary together during the PPEt practice. However, whether or not these changes were the result of causation (and what the direction of the causation might be) is unknown, although some research suggests that intentionally-directed attention to the body acts as a trigger for relaxation [39,40].

In previous research, an overestimation of duration was reported as a result of arousal due to affective states [41,42]. However, the influence of affective states on time perception seems to depend on the embodiment of emotions and to the insula, which is associated with the integration of bodily signals [43]. According to Wittmann [44] (p. 219): “The anterior insula, which integrates representations of body states with motivational and cognitive states, creates a series of ‘emotional moments’, with each moment being a coherent representation of all feelings being experienced at that time. The experience of duration develops by the integration of a series of such moments over time.” In addition, Meissner and Wittmann [45] reported a close correspondence between cardiac cycles and duration estimation. The correlation between relaxation and perception of time speed can, consequently, be thought of as a result of change in the bodily state characterized by the heart rate slowing down. The strong negative correlation of changes in body awareness with changes in time speed, as well the less strong yet significant negative correlation between changes in space awareness and changes in time speed observed in the current study both point to a similar fact, supporting the idea that integration of bodily and spatial information affects subjective experience of time passage. On the same line, we also observed a significant correlation between changes in time intensity and changes in body awareness. 

As suggested by the SMC, the change in the bodily state relates to a shift from the narrative self towards the minimal self, in which body states are integrated with motivational and cognitive states, allowing an ‘embodied experience’ in the here and now [2]. This link between the minimal self, insula and embodied experiences was also reported in a the OVO study [27] demonstrating that immersion in the OVO-WBPD chamber resulted in increased slow wave EEG activity in the insula, in parallel to increased experience of altered states of absorption (namely, feeling deeply immersed into an experience with an increased attention towards sensorial, emotional and mental contents related to that experience [46,47,48,49,50]).

Altogether, this collection of findings points to a state usually reported in deep meditative experiences, characterized by deep relaxation [47,51], which seems to affect bodily perception, which in turn affects the perception of the speed with which time passes, along with increased awareness of the experience of time [45], and time slowing down or even timelessness [8,52], in addition to disappearance of need for any agency [38], similarly to what happens in the state of overcoming of the self. Furthermore, the discursive reports provided by participants point to non-dual experiences. 

Nonetheless, the Subjective Time, Self, Space inventory questionnaires, account for participants’ state subsequently the meditation technique. As suggested by one of our Reviewers, an interpretation to the collected data is that the meditation had increased a state of mindfulness in the afterwards, in contrast to the feeling of timelessness and selflessness typical to many reports of altered states of consciousness [8,53,54]. During meditation, individuals may have experienced a diminished sense of self and time, but after meditation, when they returned to more typical states of consciousness and completed the questionnaires, they exhibited a heightened mindfulness state characterized by heightened conscious perceptions (being more present, less future oriented, feeling more intensely one’s own body, the surrounding space and the passage of time). 

In the current study we observed an increased prominence of thoughts about Present. This result is in line with studies showing that meditative practice is associated with increased awareness of the present moment [55,56]. However, our results about Past or Future were not aligned with what reported in the literature. More specifically, while we didn’t observe any change in terms of Past, and a reduction of Future, other studies showed reduced rumination related to past events or optimistic thoughts about future following mindfulness-like meditations [57,58,59]). This could be due to two main reasons. First, we tested participants after a single session of PPEt while previous results reported in the literature were mostly observed in longer protocols with at least 4 weeks of training periods. Second, in the test that we adopted, past, present and future scores were not independent from each other since their scores had to sum up to 100%. So, increasing one index would forcefully decrease other indexes. 

In light of the discussed results, the current study demonstrates, to the best of our knowledge for the first time systematically and in a large sample, a heightened state of mindfulness after a single session of meditation in non-practitioners.

### 4.2. Effects of Sex and Meditation Expertise on Space Awareness and Time Perception

We observed a significant interaction between Meditation Expertise and biological Sex in relation to Space Awareness. However, the total meditation hours experience was associated differently among males and females. More specifically, while Space Awareness scores were negatively correlated with the total meditation hours among males, the opposite was true among females. This might be due to the once widely-acknowledged greater visual spatial abilities among males compared to females ([60], although now this is considered a significant difference only in some specific subcategories of visual spatial abilities, [61]). Importantly, however, one of these specific subcategories is space awareness [62], which may be associated with greater hemispheric specialization in males compared to females [25,63,64,65]. Nevertheless, meditative practices may mitigate the effects of the hemispheric specialization by enhancing inter-hemispheric coherence [66,67,68,69,70,71], resulting in an improved hemispheric balance in both sexes reflecting an increase in spatial abilities for females and a decrease for males.

Regarding the connection between sex and the experience of time, a significant interaction between sex and total hours of meditation was observed when exploring the scores related to the saliency of the Present. More specifically, female participants showed a significant positive relation between meditation expertise (namely, for how long they practiced meditation) and Present time perspective scores, whereas males showed no such effect. Interestingly, males in our sample who had no meditation experience reported overall a tendency toward greater Present time perspective scores compared to females (M = 35.18, F = 32.06), whereas the opposite pattern was observed when comparing males and females with greater meditation experience (≥2000 h of meditation experience; M = 35.5, F = 46.57). This finding, although it is not significant, together with the fact that Zimbardo and Boyd [72,73] reported that females are more oriented towards the past and future compared to males, suggests that males’ time perspective could be considered more independent of both memories and future plans, possibly resulting in an enhanced saliency of the present moment (see also [74]). Nonetheless, this pattern appears to be affected by mindfulness-based traits. A recent study reported a stronger connection between a balanced time perspective, mindfulness-related traits and psychological well-being among a sample of female, compared to male, college students [75]. In light of this evidence, we suggest that despite males being possibly more prone to the salience of the present moment compared to females, increasing their expertise in mindfulness and/or meditation techniques that adopt awareness-based features could potentially help female practitioners in becoming more prone to the present. 

## 5. Conclusions, Limitations, and Future Studies

Our study cohort’s reports of their experiences before and after engaging in the Place of Pre-Existence (PPEt) technique suggest they generally underwent, phenomenologically speaking, a state of deep meditation, regardless of their previous meditation expertise, leading to heightened mindfulness following the meditation. This finding and the findings related to biological sex and the experience of time lends support to the conduct of further research also on sex-dependent change in the perception of time related to the PPEt and sex-dependent patterns of neural activity during this as well as other meditative techniques. Such research on sex-dependent variables could also be considered in connection to the electro-topography of selves [2]. We are currently working in these directions. 

In addition, while the focus of the current study was to examine sex-related changes in the experience of time, space and the body and the dynamic relationship between them before and after the PPEt, a major limitation of the current study is the lack of a control group which didn’t go through the PPEt. Future studies should also incorporate a passive control group. The use of neurophysiological measures to corroborate the phenomenological results, and the addition of control groups (e.g., participants practicing an alternative meditation technique, or placed on a “waitlist”) to enable comparison of their experience in connection to time, emotion and self-determination would be beneficial in future studies. In addition, more specialized studies are needed to further clarify the possible sex-related differences in relation to meditation expertise and perceptions of space and time speed. Such additional research would help elucidate the cause and nature of the seemingly lesser slowdown in time perception among females after practicing PPEt and shed light on specifically how meditation experience differentially affects the sexes regarding the perception of space after PPEt. 

Finally, although our primary experience of the world is one of a body situated within an objectively physical world, subjective interpretation plays a fundamental role in the perception of stimuli [76,77,78]. Consequently, there is a constant interplay between objective sensory stimuli and our subjective interpretation of them [12,79,80], also in terms of time perspective [81,82]. For example, a recent study of Ukrainian youth demonstrated how the 2014/2015 political crisis affected participants’ time perspectives with increasing or decreasing the negative present orientation and positive future orientation according to how directly they suffered from the situation [83]. Similarly, Kosak and colleagues [84], found how during the outbreak of COVID-19 a relative deceleration of time passage was generally associated with negative affect and social isolation; a relative acceleration was associated with an increase in routine in daily life. Indeed, a focus on the interplay between sensory stimuli and their subjective interpretation may shed new light on the relationships between the different dimensions of the self. Thus, accordingly, future studies could also examine what happens when participants undergoing troublesome experiences are guided to subjectively “collapse” the main phenomenological features of experience (e.g., time, emotion, and self-determination). It is possible that through a phenomenological “collapse” participants might re-narrate the self by moving towards the minimal self and the overcoming of the self and consequently reshape their time perspective. 

## Data Availability

Data will be available on request.
